# Role of Physical Activity in Bone–Muscle Crosstalk: Biological Aspects and Clinical Implications

**DOI:** 10.3390/jfmk6020055

**Published:** 2021-06-21

**Authors:** Ida Cariati, Roberto Bonanni, Federica Onorato, Ambra Mastrogregori, Danilo Rossi, Riccardo Iundusi, Elena Gasbarra, Virginia Tancredi, Umberto Tarantino

**Affiliations:** 1 PhD in Medical-Surgical Biotechnologies and Translational Medicine, “Tor Vergata” University of Rome, Via Montpellier 1, 00133 Rome, Italy; ida.cariati@uniroma2.it; 2 Department of Clinical Sciences and Translational Medicine, “Tor Vergata” University of Rome, Via Montpellier 1, 00133 Rome, Italy; 3 Department of Systems Medicine, “Tor Vergata” University of Rome, Via Montpellier 1, 00133 Rome, Italy; roberto.bonanni1288@gmail.com (R.B.); tancredi@uniroma2.it (V.T.); 4 Department of Orthopaedics and Traumatology, “Policlinico Tor Vergata” Foundation, Viale Oxford 81, 00133 Rome, Italy; fede.onorato@gmail.com (F.O.); ambra.mastrogregori1993@gmail.com (A.M.); dnlrss92@gmail.com (D.R.); riccardo.iundusi@uniroma2.it (R.I.); gasbarra@med.uniroma2.it (E.G.); 5 Centre of Space Bio-Medicine, “Tor Vergata” University of Rome, Via Montpellier 1, 00133 Rome, Italy

**Keywords:** bone–muscle crosstalk, osteosarcopenia, myokines, osteokines, physical activity, prevention strategy

## Abstract

Bone and muscle tissues influence each other through the integration of mechanical and biochemical signals, giving rise to bone–muscle crosstalk. They are also known to secrete osteokines, myokines, and cytokines into the circulation, influencing the biological and pathological activities in local and distant organs and cells. In this regard, even osteoporosis and sarcopenia, which were initially thought to be two independent diseases, have recently been defined under the term “osteosarcopenia”, to indicate a synergistic condition of low bone mass with muscle atrophy and hypofunction. Undoubtedly, osteosarcopenia is a major public health concern, being associated with high rates of morbidity and mortality. The best current defence against osteosarcopenia is prevention based on a healthy lifestyle and regular exercise. The most appropriate type, intensity, duration, and frequency of exercise to positively influence osteosarcopenia are not yet known. However, combined programmes of progressive resistance exercises, weight-bearing impact exercises, and challenging balance/mobility activities currently appear to be the most effective in optimising musculoskeletal health and function. Based on this evidence, the aim of our review was to summarize the current knowledge about the role of exercise in bone–muscle crosstalk, highlighting how it may represent an effective alternative strategy to prevent and/or counteract the onset of osteosarcopenia.

## 1. Introduction

Over the past decade, knowledge about the secretory activity of bone and muscle has been greatly improved. It is now accepted that both tissues are responsible for the secretion of a wide variety of molecules with hormonal function, as well as influencing each other through mechanical stress [1,2,3]. These mechanical and biochemical inputs should not be considered as separate signals, but as two important factors cooperating synergistically in the same system. Indeed, mechanical stresses regulate the production of osteokines and myokines with autocrine, paracrine, and endocrine functions; in turn, these induce molecular and structural changes in cells and tissues, improving their ability to respond to mechanical stress [4,5]. The production and release of these signal molecules is the basis of communication between the two tissues, which is currently known as bone–muscle crosstalk [6,7]. However, the underlying mechanisms have not yet been fully elucidated.

**Figure 1:**
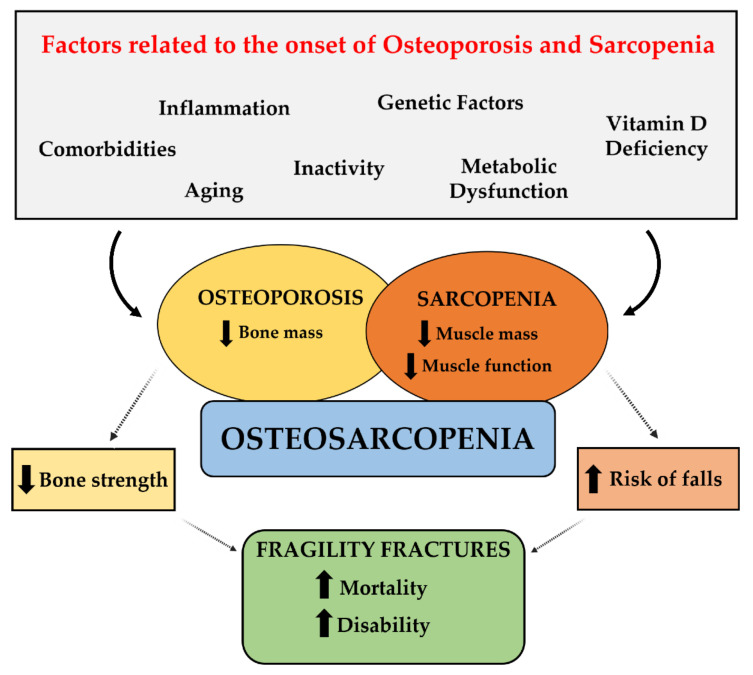
Pathophysiology of osteosarcopenia.

Because bone and muscle are two parts of the same unit, diseases involving these tissues should be considered interrelated and interdependent rather than treated independently of each other. For example, osteosarcopenia is a newly defined geriatric syndrome, resulting from the combination of osteoporosis and sarcopenia, which are two widespread conditions with high mortality and morbidity [8,9] (Figure 1). Like other medical conditions, osteosarcopenia is a major and growing global health concern, so the focus should be on preventing its occurrence rather than treating it [10]. Indeed, its complex and multifactorial nature requires multifaceted treatment and prevention strategies. A healthy lifestyle and regular exercise are the first-line choices for the prevention and treatment of osteosarcopenia [11].

Based on this evidence, the aim of our review was to investigate the impact of exercise on bone and muscle tissue health by (i) summarizing the current knowledge on the mechanical and biochemical interactions underlying bone–muscle crosstalk; and (ii) highlighting how exercise may represent an effective alternative strategy to prevent and/or counteract the onset of osteosarcopenia.

## 2. Literature Search Strategy

For this narrative review, 128 papers were selected from the Medline bibliographic database, published between 1945 (starting date) and 2021. Papers concerning the mechanical and biochemical interactions between bone and muscle tissues and the role of physical activity in bone–muscle crosstalk and osteosarcopenia were included. The search strategy was based on the use and/or combination of the following keywords: “bone-muscle crosstalk”; “mechanical interactions”; “biochemical interactions”; “osteokines”; “myokines”; “osteoporosis”; “sarcopenia”; “osteosarcopenia”; “fragility fractures”; “physical activity”; “exercise”. The search process was performed on a worldwide basis, without excluding specific geographic areas or different ethnic groups. Language and species filters were applied to the results list to eliminate non-English articles.

## 3. Bone–Muscle Crosstalk: Mechanical and Biochemical Aspects

The bone–muscle system is extremely complex, and an individual analysis of its components may not be sufficient for a complete understanding of the functions shared by the two tissues. Communication between bone and muscle tissues depends on both biochemical interactions and mechanical stresses. Indeed, the two tissues influence each other through the secretion of hormone-acting molecules. In addition, as skeletal muscles apply forces to the bone tissue, it in turn provides support and anchorage to the muscles [12]. Therefore, the complexity of the bone–muscle crosstalk depends on the mutual biochemical–mechanical influence between the tissues.

### 3.1. Mechanical Aspects

For normal maintenance of a vertical body position, the skeleton provides rigid levers to the muscles on which they apply forces of varying intensity, inducing structural deformations [13]. This mechanical interaction between bone and muscle, which depends on the mechanotransduction process, involves the conversion of mechanical forces into intracellular molecular signals, leading to changes in the cytoskeleton and cellular communication [14]. Bone mass can also be affected by mechanical loading through the involvement of cellular mechanisms that regulate bone remodelling [15,16]. Therefore, physical therapy, which involves increasing muscle strength, is an effective tool to improve bone health [17].

Osteocytes have been reported to be potential players in the perception of mechanical stimuli, being able to sense load through different mechanisms, such as physical deformation of the bone matrix [18]. Although it has been reported that these cells can perceive mechanical forces through the cell body or dendritic processes, the underlying mechanisms are still under investigation [19]. Integrins, cell adhesion proteins that interact with extracellular matrix ligands, have been suggested to play an important role in the transfer of forces applied to the cell membrane to distant compartments, such as the nucleus or mitochondria [20]. Particularly, integrins might act in association with ion channels that respond to stimuli of various types, such as changes in tension, stretch, and flow-related shear stress. So, cellular stretching could result in mechanical activation of these channels and induce a subsequent change in intracellular chemical gradients [19].

Sclerostin, an osteocyte-specific protein, has also been suggested as a key molecule in mechanotransduction, being able to integrate mechanical, local, and hormonal signals detected by osteocytes. Specifically, it has been observed that the absence of loading increases the levels of sclerostin, promoting bone resorption, while the increase in load reduces its levels in favour of bone formation [21]. Despite the important role played by sclerostin in bone remodelling and integration of various signals, the involvement of other important osteocyte-derived mediators in mechanotransduction cannot be excluded. For this reason, the identification and understanding of the molecular mechanisms underlying the conversion of mechanical stimuli into biochemical signals is currently a major challenge, which could provide additional tools to improve the therapeutic treatment of the musculoskeletal system disease. In this regard, Judex et al. evaluated the effect of catabolic (disuse) and anabolic (45 Hz, 0.3 g vibration) signals on the mRNA levels of a group of genes whose expression plays a key role in bone formation [22]. By means of histomorphometric analysis of the tibial metaphysis and periosteal and endocortical surfaces of the diaphysis, the authors assessed bone formation rates, finding a 55% increase in mice exposed to the mechanical signal and a 60% decrease in mice subjected to disuse. However, despite significant differences in bone formation rates, the expression pattern of the 13 genes selected by the authors did not change significantly over the course of the experiment, suggesting that the conversion of mechanical stress into intracellular signals is complex and finely regulated [22].

Interestingly, several scientific studies have suggested the existence of a correlation between mechanical stimuli, muscle mass, and protein synthesis. Enhanced mechanical loading has been reported to induce changes in muscle mass and increase protein synthesis, thus causing hypertrophy [23]. The serine–threonine kinase mechanistic Target of Rapamycin (mTOR) has been suggested as a key element in this correlation, as its activation could trigger the mechanisms underlying load-induced muscle hypertrophy [23]. This serine–threonine kinase is present in both mTOR complex 1 (mTORC1) and mTOR complex 2 (mTORC2) [24]. Notably, mTORC1 signalling is of critical importance for the regulation of protein synthesis and its activation depends on a wide range of mechanistic stimuli, which, however, have not yet been elucidated [25]. Insulin-like Growth Factor-1 (IGF-1) might be among those responsible for mTORC1 activation. Indeed, overexpression of IGF-1 has been shown to promote activation of the PI3K/Akt/mTORC1 pathway, inducing increased protein synthesis and muscle hypertrophy [26,27].

Finally, further confirmation of the importance of mechanical stress on the function of the bone–muscle system has been provided by simulated microgravity studies, which have shown that spaceflight induces muscle atrophy, resulting in loss of bone mass [28]. Specifically, it has been hypothesized that long-term exposure to simulated microgravity may affect tissue-specific adult stem cell proliferation and differentiation, with a negative impact on normal tissue growth and regenerative repair [29,30]. In this regard, we have recently investigated the role of Bone Morphogenetic Protein-2 (BMP-2) and myostatin in the response of human satellite cells from osteoporotic, osteoarthritic, and healthy patients to simulated microgravity conditions, to identify the main molecules involved in the degeneration/regeneration phenomena of muscle tissue related to altered mechanical loading [31]. We observed that the simulated microgravity regime affected the primary satellite cell cultures not only morphologically but also from a molecular point of view, causing a significant increase in BMP-2 expression in all experimental conditions and a significant reduction in myostatin expression only in osteoporotic patients. These results allowed us to confirm that the changes observed in astronauts during spaceflight are reminiscent of the onset and progression of major musculoskeletal disorders, such as osteoporosis and sarcopenia, suggesting the likely involvement of common physiological mechanisms [31].

### 3.2. Biochemical Aspects

All cellular elements responsible for bone tissue metabolism, including osteoblasts, osteocytes, osteoclasts, chondroblasts, and chondrocytes, act under the influence of muscle, underlining the key role of this tissue in defining bone quality [32]. Furthermore, several scientific studies agree that both tissues perform an important endocrine function, whose products, represented by osteokines for bone tissue and myokines for muscle tissue, have been proposed as possible key players in bone–muscle crosstalk [2,6,33]. Levels of these signal molecules can also vary with age and physical activity [34]. Therefore, the identification of these molecules and understanding their role within the bone–muscle system is currently a major challenge to identify potential pharmacological targets and to prevent and/or treat bone–muscle diseases related to aging and sedentariness.

## 4. Osteokines and Myokines: Two Players on the Same Team

### 4.1. Osteokines

The idea that bone has endocrine functions that can influence the activity of other organs and, more generally, energy metabolism, is increasingly shared. This surprising function of bone tissue is expressed through the secretion of signal molecules with hormonal function known as osteokines (from the Greek “osteo” meaning “bone” and “kino” meaning “movement”), which are factors derived from bone cells influencing local and systemic metabolism [35]. Unlike skeletal muscle, from which more than 600 myokines have been identified, bone has only been recognized as an endocrine organ since 2007, so research regarding bone-derived factors that mediate the bone–muscle crosstalk is still limited [36].

Among the most studied molecules for which a role of osteokines has been suggested, we focused our attention on Osteocalcin (OCN), sclerostin, and Fibroblast Growth Factor 23 (FGF23), as well as on the Receptor Activator of Nuclear Factor Kappa B (RANK)/RANK Ligand (RANKL)/Osteoprotegerin (OPG) pathway [37]. Although the topic is of relevant importance, most of the information in the literature refers to in vitro and in vivo studies, thus suggesting the need for further research to understand the action of these signal molecules in humans.

#### 4.1.1. OCN

OCN is a hormone secreted mainly by osteoblasts and is present in the circulation in carboxylated, undercarboxylated, and non-carboxylated forms [2]. The increasing number of functions attributed to OCN raises the question of whether circulating levels of this hormone change in various physiological situations, including regulation of processes severely affected by aging and muscle function during exercise [38,39].

Clinical studies have shown that non-carboxylated or undercarboxylated forms of OCN (ucOCN) increase after exercise in young and elderly subjects [40,41,42]. Although the hormonal action of OCN on skeletal muscle is still unclear, it has been reported that (i) undercarboxylated OCN causes an insulin-dependent increase in post-contraction glucose uptake; and (ii) mice lacking OCN show a reduction in muscle mass, indicating the complex role of this molecule in bone–muscle crosstalk [2].

Similarly, Mera et al. suggested that OCN influences muscle contractility and mitochondrial biogenesis in myofibers of young adult mice during exercise by (i) promoting glycogen breakdown, thus contributing to the supply of glucose that is required for muscle contraction during exercise; (ii) inducing translocation of the Glucose Transporter Type 4 (GLUT4) to the plasma membrane, which results in enhanced glucose uptake and glycolysis; and (iii) increasing fatty acids uptake and catabolism. Taken together, all these functions promote the activity of the Tricarboxylic Acid Cycle (TCA) and thus the production of Adenosine Triphosphate (ATP) required to increase muscle function [43].

ucOCN would also appear to be involved in muscle hypertrophy and strength; indeed, mice with OCN deletions have lower muscle mass, and ucOCN administration increased the muscle mass in older mice [43,44,45]. It has also been suggested that bone–muscle crosstalk might depend on a mechanism involving OCN and IL-6 (Interleukin-6) signalling simultaneously [2], as significant increases in both muscle-derived IL-6 and ucOCN post-endurance exercise were found. In addition, IL-6-deficient mice did not show the typical increase in OCN post-exercise, indicating that the chemokine was required for this crosstalk [46].

Finally, OCN has been reported to be sufficient to reverse the decline in muscle function that occurs during aging, as its administration increased the exercise capacity of 3-month-old wild-type mice and restored the exercise capacity of 9-, 12-, and even 15-month-old mice. Taken together, these results suggest OCN signalling in myofibers as a novel and powerful means to combat age-related decline in muscle function [43].

#### 4.1.2. Sclerostin

Sclerostin is a circulating protein produced by osteocytes that inhibits the Wnt/beta-catenin signalling pathway, playing a central role in insulin resistance, inflammation, and metabolic disorders [47,48]. Wnt signalling is known for its pivotal role in osteoblast differentiation and suppression of osteoclastic development, as demonstrated by experiments in mice, in which downregulation or neutralization of the Wnt antagonists improved bone formation [49]. The importance of Wnt signalling in bone formation is indicated by skeletal disorders, such as sclerosteosis, which is characterized by increased osteoblast activity [50,51]. The absence of sclerostin, caused by the loss of the *SOST* gene, appears to play a crucial role in the onset of this disease. Noteworthy, Phase II clinical trials demonstrated that one year of treatment with anti-sclerostin antibodies promoted bone formation, reduced bone resorption, and significantly increased Bone Mineral Density (BMD) [49].

The idea that sclerostin acts as a negative regulator of bone formation derives from studies in mice and humans demonstrating its ability to inhibit the differentiation and mineralization of murine preosteoblastic cells, as well as significantly impair the Alkaline Phosphatase (ALP) activity and calcium deposit of osteoblastic cells treated with sclerostin [52].

A better understanding of the mechanisms underlying mechanical stress-induced *SOST*/sclerostin regulation was provided by Robling et al. using two different mouse models, one with enhanced ulnar axial loading and one with hindlimb unloading [53]. Mice subjected to ulnar loading showed a significant reduction in sclerostin-positive osteocytes of about 15% in the proximal section of the diaphysis, which undergoes minor loading peaks, and of about 60% in the distal section, which is more stressed by loading. Notably, the load-induced reduction in sclerostin levels was correlated with a reduction in *SOST* gene mRNA. In contrast, in mice subjected to hindlimb unloading, no significant difference in sclerostin-positive osteocytes was found. Thus, the authors concluded that the sclerostin levels could be finely regulated through a mechanism in which osteocytes coordinate osteogenesis in response to increased mechanical stimulation [53].

Similarly, the effects of loading on sclerostin expression were also observed by Moustafa and colleagues, who subjected the right tibia of 19-week-old female mice to non-invasive dynamic axial loading and/or disuse by sciatic neurectomy [54]. Immunohistochemical analysis showed that mechanical loading was correlated with decreased staining of sclerostin-positive osteocytes and increased both bone formation and bone volume. Conversely, disuse resulted in an increase in the percentage of sclerostin-positive osteocytes. However, the downregulation of the load-induced sclerostin was not uniform throughout the bone and appeared to be more associated with subsequent bone formation rather than the magnitude of the peak deformation generated by the load [54].

Recently, Kim and colleagues correlated serum sclerostin levels with muscle mass in 240 healthy nondiabetic subjects to investigate the involvement of sclerostin in their low muscle mass [55]. Serum sclerostin levels were observed to be negatively correlated with skeletal muscle mass, independent of confounding factors such as age, sex, BMD, and total fat mass, suggesting a possible role for this osteokine as a marker of low muscle mass [55]. In this regard, Magarò et al. observed sclerostin expression both in muscle cells in vitro and in muscle taken from mice at different ages, indicating for the first time skeletal muscle as a new source of sclerostin [56]. This surprising discovery brings the complexity of the bone–muscle crosstalk to a higher level, as sclerostin produced in muscle could act synergistically with that produced in bone and exacerbate the state of fragility typical of pathological conditions characterized by simultaneous loss of bone and muscle, such as osteosarcopenia [56].

#### 4.1.3. RANK/RANKL/OPG

The delicate balance between bone formation, through the activity of osteoblasts, and bone resorption, operated by osteoclasts, is strictly dependent on the RANK/RANKL/OPG pathway [57,58]. RANK is a transmembrane homotrimer receptor expressed in mature osteoclasts, osteoclast precursors, dendritic cells, and mammary glands; in turn, RANKL is a membrane-bound homotrimer protein of activated T cells and osteoblasts, but can also be secreted as a result of proteolytic cleavage or alternative splicing [59]. It is known that the formation, activation, and survival of osteoclasts in normal bone modelling and remodelling depend on RANK/RANKL signalling, as well as several pathological conditions characterised by increased bone turnover. In contrast, OPG has a protective effect on bone by binding to RANKL and preventing its interaction with RANK [59].

RANK is also expressed in skeletal muscle and the activation of the RANK/RANKL pathway leads to inhibition of myogenic differentiation through activation of Nuclear Factor kappa-B (NF-κB), resulting in skeletal muscle dysfunction and loss [60]. Indeed, it was recently observed that RANK/RANKL levels were increased by about twice as much in dystrophic mice and that anti-RANKL treatment improved muscle function by inhibiting muscle degeneration [61]. Similarly, Hamoudi et al. observed that OPG-deficient mice showed signs of muscle weakness, atrophy of rapidly contracting type II b myofibers, and increased expression of atrophic proteins [62].

The RANK/RANKL/OPG signalling has been proposed to be modulated by physical activity, although conflicting information currently exists. Some evidence suggests that exercise can modulate the RANK/RANKL/OPG pathway with consequent beneficial effects on bone health [63]. The results of a study conducted on rats with glucocorticoid-induced osteoporosis found that training on a treadmill and vibrating platform significantly decreased the RANKL expression and increased the OPG levels, improving the osteoporotic condition [64]. Thus, exercise can modulate RANK/RANKL/OPG signalling, exerting beneficial effects on bone tissue health.

#### 4.1.4. FGF23

FGF23 is a 23 KDa glycoprotein of the Fibroblast Growth Factor (FGF) superfamily, mainly synthesized by osteocytes in adult trabecular bone [65,66]. It is an important regulator of serum phosphate levels, as it increases phosphate excretion by controlling the expression and insertion of sodium-phosphate transporters into renal proximal tubule membranes [67]. In addition, FGF23 is known to inhibit renal expression of 1alpha hydrolase, contributing to reduced intestinal and bone absorption of phosphate [67]. It is also involved in inflammation, erythropoiesis, and iron economy [68]. Because of its multiple roles, FGF23 has been proposed as the major endocrine factor secreted by osteocytes [69]. The importance of this glycoprotein in bone health is not new, as it appears to be involved in tumour-induced osteomalacia [70,71] and osteoporosis [72]. In addition, FGF23 knockout mice exhibit some typical effects of aging, such as a reduced lifespan, cognitive impairment, and cardiac hypertrophy [73].

The action of FGF23 on skeletal muscle is still poorly understood. In 2016, Li et al. provided important evidence on exercise-induced FGF23 production. Specifically, the authors studied the effects of exposing C57BL/6J mice to three forms of exercise (acute, strenuous, and moderately chronic) by analysing serum FGF23 concentrations and its expression in skeletal muscle [74]. The effect of treatment with recombinant FGF23 on exercise endurance, intramuscular levels of Reactive Oxygen Species (ROS), and markers of mitochondrial function was also evaluated. All three types of exercise significantly increased the serum levels of the glycoprotein, but chronic exercise only moderately improved FGF23 mRNA expression in skeletal muscle. Furthermore, FGF23 administration induced greater exercise endurance and significantly reduced the ROS levels in skeletal muscle. These results indicate the existence of a correlation between increased muscle activity and FGF23 production, suggesting an important role for this glycoprotein in skeletal muscle health [74]. However, skeletal muscle dysfunction has been found in patients with hereditary hypophosphatemic rickets related to chronic serum accumulation of FGF23 [75,76]. In this regard, Avin et al., evaluating the changes that occurred in the muscle of mouse models after FGF23 treatment, found no changes in terms of myogenesis, oxidative stress, intracellular calcium concentration, and myostatin expression [77]. Therefore, it has been suggested that FGF23 alone is not able to induce changes in skeletal muscle, but that other substances are needed to act together with this factor.

### 4.2. Myokines

Myokines are cytokines synthesized and secreted by myocytes in response to muscle contraction. They perform an autocrine function, regulating muscle metabolism, and an autocrine/paracrine function, acting on other distant tissues and organs [1]. The currently identified myokines, thanks to the development of modern sequencing and analysis technology, are mainly composed of peptides, such as growth factors, cytokines, and some small organic acids, and all interact with each other to maintain homeostasis through muscle–organ crosstalk [78,79]. Therefore, we have focused on the most studied myokines, which are involved in metabolism and health of bone and skeletal muscle.

#### 4.2.1. Myostatin

Myostatin, also known as Growth Differentiation Factor 8 (GDF8), is a protein belonging to the Transforming Growth Factor-β (TGF-β) superfamily produced by skeletal muscle, on which it acts as a negative regulator [34,80]. Myostatin deficiency is associated with increased muscle mass and strength, suggesting the strong impact this myokine may have in bone–muscle crosstalk. Myostatin-deficient mice are characterized by an expansion of muscle insertion sites in the humerus, femur, and spine. Specifically, in the proximal humerus, an increase in trabecular area and Bone Mineral Content (BMC) was observed, and in the femur, an increase in both shaft diameter and BMD was detected [81].

The anti-osteogenic role of this myokine was also discussed by Qin et al., who observed that, in response to myostatin, osteocytes produce large amounts of sclerostin, RANKL, and Dickkopf-1 (DKK-1), which are important regulators of bone remodelling [82].

Myostatin expression is known to downregulate by exercise [83]. Analysis of large muscle biopsies from haemodialysis patients revealed that nine weeks of endurance cycling exercise halved the myostatin mRNA levels. In addition, even following a single period of resistance training, a significant reduction in myostatin mRNA was observed in gastrocnemius biopsies in physically active men and women, suggesting a close correlation between exercise and myostatin expression [84]. Finally, it has recently been reported that the Androgen Receptor (AR), whose muscle production increases with exercise, transcriptionally inhibits myostatin expression [85]. Therefore, through regulation of myostatin, exercise could modulate bone–muscle crosstalk and counteract the onset of aging-related diseases.

#### 4.2.2. Interleukins

Interleukins are inflammatory mediators secreted by a variety of cell types throughout the body, including skeletal muscle cells, and play an important role in bone–muscle crosstalk. For example, it has been reported that circulating levels of Interleukin-15 (IL-15), which has an anti-atrophic function, increase in response to resistance exercise in both trained and untrained subjects [57]. A correlation between the IL-15 receptor α (IL-15Rα) and osteogenic cell function has also been documented. In fact, the absence of IL-15Rα decreased bone mineralization in vivo and in vitro, and IL15Rα^-/-^ osteogenic cells showed a reduced RANKL/OPG mRNA ratio, highlighting a defect in osteoblast–osteoclast coupling [58].

One of the most studied interleukins is undoubtedly Interleukin-6 (IL-6), which is released from muscle in response to exercise and contraction, acting as an anti-inflammatory compound and increasing glucose uptake and sensitivity [46,86,87]. The increased circulating levels of IL-6 during exercise has recently been reported to promote exercise capacity. This feedback mechanism involves bone tissue, as IL-6 triggers signalling in osteoblasts leading to the production of RANKL, which in turn induces osteoclast differentiation and subsequent OCN release [46]. However, IL-6 is also known for its pro-inflammatory role and can activate different signalling pathways, depending on the receptor involved. It has been defined as a pleiotropic cytokine and deregulation of its signalling pathways can lead to the development of inflammatory, autoimmune, and cancer diseases [88]. Therefore, inhibition of IL-6, or the signalling pathways in which it is involved, by therapeutic agents represents a very active area to counteract the progression of several diseases [88].

Finally, there is scientific evidence that other interleukins are also produced during exercise, such as Interleukin-7 (IL-7) and Interleukin-8 (IL-8), which are strongly related to inflammatory responses and expressed during muscle contraction, and Interleukin-10 (IL-10), whose increase in plasma levels correlates with the duration of exercise [89,90,91].

#### 4.2.3. Irisin

Irisin is a myokine secreted in abundance by skeletal muscle in response to exercise, in both mice and humans. After its release into the circulation, irisin acts on white adipocytes to induce the browning response and thereby activate shiver-free thermogenesis [92]. However, multiple irisin-induced effects on bone tissue have also been recently documented. Colaianni et al. demonstrated not only that irisin promotes osteoblast differentiation in vitro, but also that osteoblasts increased the expression of ALP and Collagen I in an irisin-dependent manner [93]. Furthermore, the injection of low doses of recombinant irisin into young male mice was observed to induce significant increases in BMD of cortical tissue, periosteal circumference, and flexural strength, indicating irisin-induced bone stimulation [94]. Noteworthy, it was shown that treatment with r-Irisin could both prevent and recover bone loss achieved by keeping mice with their hind limbs suspended, demonstrating the efficacy of this myokine as a preventive and curative agent [95].

Recently, a role for irisin in bone remodelling has also been proposed, as it not only promoted the sclerostin production [96] but could directly regulate osteoclasts by increasing differentiation of mouse bone marrow progenitors [97]. Although the regulatory effects of irisin on bone metabolism are still debated, there is promising evidence for its beneficial effects in bone–muscle crosstalk.

#### 4.2.4. ΒAIBA

Beta-Aminoisobutyric Acid (BAIBA) is a small molecule (103.6 Da) consisting of two enantiomers, L-BAIBA and D-BAIBA, produced by skeletal muscle during exercise [98]. It acts endocrinally on various tissues and is therefore considered one of the mediators of the beneficial effect of exercise from skeletal muscle on the body [99]. BAIBA is known to activate the β-oxidative pathway of hepatic fatty acids and trigger browning of white adipose tissue, as well as improving insulin resistance and inflammation in skeletal muscle through an autocrine/paracrine action [98,100]. It also prevents diet-induced obesity and protects against metabolic disorders in type 2 diabetes [101,102].

Roberts et al. have suggested a role for BAIBA also as a bone-protective factor, since this myokine appears to prevent ROS-induced apoptotic death of osteocytes through binding to the Mas-related G protein-coupled receptor type D (MRGPRD). However, this protective capacity seems to be lost with aging due to the under-regulation of MRGPRD expression in osteocytes [98].

Finally, the beneficial effects of BAIBA were also observed in vivo. Hindlimb unloading experiments were performed by keeping mice with their limbs elevated for a fortnight [99]. Surprisingly, mice that received L-BAIBA in drinking water showed a significantly greater bone volume fraction and trabecular bone thickness than the control groups. These results suggest the need for more in-depth studies to correct the reduced skeletal response to exercise with aging and to better understand the mechanisms underlying bone–muscle crosstalk during aging [99].

**Figure 2:**
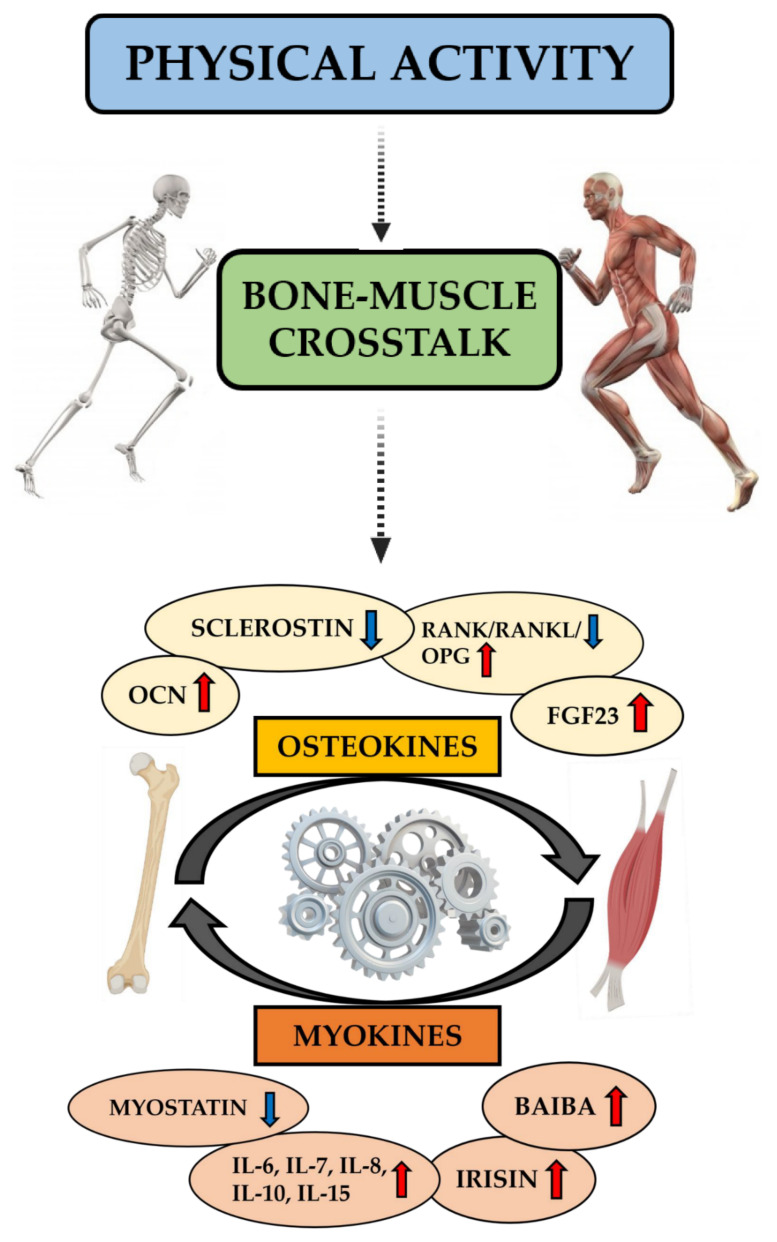
Role of physical activity in bone–muscle crosstalk. Physical activity plays a central role in bone–muscle crosstalk and the health of these tissues by regulating osteokine and myokine production and optimizing mechanical stress. During exercise, bone production of Osteocalcin (OCN), Osteoprotegerin (OPG), and Fibroblast Growth Factor 23 (FGF23) increases, whereas levels of Receptor Activator of Nuclear Factor Kappa B (RANK)/RANK Ligand (RANKL) and sclerostin are reduced. On the other hand, physical activity stimulates muscle tissue to produce Interleukins (IL-6, IL-7, IL-8, IL-10, and IL-15), irisin, and Beta-Aminoisobutyric Acid (BAIBA), whereas myostatin secretion is reduced. Mechanical and biochemical signals ensure communication between bone and muscle tissues.

Figure 2 summarizes the role of physical activity in bone–muscle crosstalk, showing how bone and muscle tissues interact not only mechanically but also through the exchange of biochemical signals. Specifically, these are osteokines and myokines, the expression of which increases or decreases in the circulation following exercise, with short- and long-term effects on local and distant organs and tissues.

## 5. Bone–Muscle Crosstalk and Osteosarcopenia: Pathological and Clinical Features

The term “osteosarcopenia” has recently been coined to indicate the coexistence of osteoporosis and sarcopenia, the two main chronic musculoskeletal disorders associated with the aging process [103].

Osteoporosis is defined as a systemic skeletal disease characterized by reduced bone mass and qualitative skeleton changes, resulting in increased bone fragility and enhanced risk of fractures [104,105]. On the other hand, sarcopenia is a generalized pathological condition consisting of age-related loss of muscle mass and function; it is strongly associated with reduced physical strength and poor quality of life, as the patient is at increased risk of falls and fractures and is bedridden with an increased risk of mortality [106,107].

Although the mechanisms causing the loss of bone and muscle mass are still unclear, it is now accepted that a decline in muscle function causes a decrease in bone load, which results in bone loss [108]. However, the reduction in bone mass does not completely explain the onset of sarcopenia, nor does muscular atrophy explain all osteoporosis [13].

The mechanical and biochemical nature of the bone–muscle crosstalk largely confirms that bone and muscle tissues are closely linked to each other and, when the aging process starts to affect one of them, the functionality of the other is also affected [109]. From this point of view, osteoporosis and sarcopenia are two sides of the same coin, that of bone fragility, since they often coexist in a frail subgroup of the elderly population, leading to significantly worse outcomes than those observed in either condition alone [30].

Since bone and muscle are now considered as a single unit, osteoporosis and sarcopenia should be considered simultaneously in the treatment of frail patients [11,110]. Thus, bone–muscle interactions, which are the basis of crosstalk, should be the focus for the development of diagnostic and therapeutic strategies to improve the quality of the bone–muscle axis [7]. In this regard, a major challenge is to understand the role of “osteo-myokines” and how their expression is regulated by mechanical stimuli between bone and muscle.

In recent years, the attention of many researchers has focused on irisin, which has been positively correlated with BMD and negatively correlated with age, highlighting the centrality of its role in bone–muscle crosstalk and the importance of exercise in the health of the bone–muscle system [111]. However, the great complexity of this system and the large number of osteo-myokines involved in the bone–muscle crosstalk makes it difficult to identify a unique and specific target for osteosarcopenia, whose pathogenesis is multifactorial and depends not only on mechanical and biochemical factors, but also on genetic and lifestyle factors that contribute to the involution of the bone–muscle unit [112].

Notably, osteosarcopenia is a condition of increasing importance with significant negative consequences both for patients, who are exposed to increased morbidity, mortality, and disability, and for society, given the enormous socio-economic burden to which it is subjected. Thus, a better understanding of the interactions between the two tissues could facilitate the development of new therapeutic agents directed at the bone–muscle unit, in addition to nutritional and exercise-based therapies that would allow a more holistic approach to osteosarcopenia in the future [103].

## 6. Physical Activity as Preventive Strategy for Osteosarcopenia

The best current defence against osteosarcopenia is prevention. In fact, it is well known that bone is a dynamic tissue that responds to a variety of physical stimuli, including movement, traction, and vibration, which enable locomotion and are fundamental in bone and muscle remodelling [113]. The most appropriate type, intensity, duration, and frequency of exercise to positively influence osteosarcopenia are not yet known [114].

Resistance exercises are known to substantially reduce the loss of bone and muscle mass associated with age, as well as provide overall benefits to the entire body [115]. Specifically, it has been suggested that Dynamic-Resistance Exercise (DRT), supported by adequate dietary supplementation, could be the most promising strategy to improve the clinical conditions of elderly osteosarcopenic patients, with beneficial metabolic, nervous, and cardiovascular effects [116]. Based on this evidence, Kemmler et al. investigated the effect of High-Intensity Dynamic Resistance Exercise Training (HIT-RT) combined with the administration of milk proteins, calcium, and vitamin D in older men with osteosarcopenia. Indeed, the authors observed an increase in bone and muscle quality, concluding that endurance resistance, in combination with a nutritional intervention, represents a safe and effective option for the treatment of age-related osteosarcopenia [117]. Similarly, Watson and colleagues examined the safety and efficacy of 8-month High-intensity Progressive Resistance Training (HiPRT) in postmenopausal women older than 60 years with low to very low bone mass [118]. Surprisingly, benefits were found with only two 30-min training sessions per week in bone mass, lean and fat mass, physical function, and stature. So, the authors proposed HiPRT as a safe and effective type of training to improve bone strength indices and fracture risk in postmenopausal women [118].

Not all forms of exercise are equally effective in improving the quality of the bone–muscle system. Progressive Resistance Training (PRT) certainly has a positive impact on muscle mass, size, and strength, as well as on bone health, while regular but low-intensity physical activity (walking) is not sufficiently effective in improving osteosarcopenic conditions [119]. In fact, it has been reported that walking does not significantly improve BMD in pre- and postmenopausal women, suggesting that this form of exercise is not an appropriate approach to counteract osteopenia [120]. In contrast, resistance training protocols, such as running, have been shown to be highly effective in preserving and improving BMD in pre- and postmenopausal women [121]. Similarly, progressive strength training has been demonstrated to benefit the muscular health of elderly sarcopenic patients by improving the strength and performance of simple and complex physical tasks [122]. Finally, a recent meta-analysis investigated the role of exercise on sarcopenia-related outcomes, reporting improved muscle mass, strength, and physical function [123].

Resistance exercise has also been suggested to stimulate the complex processes involved in mechanotransduction and myokine production, with endocrine, autocrine, and paracrine functions acting synergistically causing muscle hypertrophy through protein synthesis [124]. In addition, performing resistance exercises three times a week for 12–24 weeks has been shown to prevent loss of muscle mass in obese elderly people on a low-calorie diet [125].

Finally, Whole Body Vibration (WBV), a process in which a vibrating force is transmitted to muscles and bones, has been proposed to play a positive role in both osteopenia and sarcopenia. However, the effects of WBV on BMD are contradictory, probably due to the lack of an adequate protocol in terms of optimal vibration frequency and duration of therapy [126,127,128].

In conclusion, targeted multimodal programmes combining traditional and high-speed PRT, weight-bearing impact exercises, and challenging balance/mobility activities appear to be currently the most effective in optimizing musculoskeletal health and function [119].

## 7. Discussion

Bone and muscle function are an integral part of locomotion, both of which are affected by advancing age. In according to the concept of “bone–muscle unity”, there is communication between both tissues; thus, a disease affecting one part of the musculoskeletal unit is likely to affect the other, and vice versa.

Mechanical and metabolic relationships between bone and muscle have been identified, as well as common mechanisms involved in the development of osteoporosis and sarcopenia. However, the association between biochemical and mechanical signals underlying bone–muscle crosstalk is not yet fully understood. The identification of molecular targets that perceive mechanical stress and respond by regulating protein synthesis and secretion could represent a major challenge for the development of therapeutic approaches directed against bone–muscle diseases.

Among these, osteosarcopenia is a growing global health concern, as its complex and multifactorial nature requires multifaceted treatment and prevention strategies. A healthy lifestyle and regular exercise are currently the first-line choices for the prevention and treatment of osteosarcopenia. Indeed, regular physical activity has been shown to significantly improve the health of the bone–muscle system, suggesting a role for exercise in preventing and/or delaying the development of osteosarcopenia. In addition, constant and appropriately designed training could improve bone–muscle crosstalk by regulating and intensifying the exchange of mechanical and biochemical signals between the two tissues.

The molecular mechanisms involved in exercise-induced changes in bone–muscle crosstalk are multiple and still unclear. Further studies to better understand the mechanical and biochemical signals triggered by exercise could be the starting point for the development of new strategies to improve the quality of life of osteosarcopenic patients and to prevent and/or delay the onset of major age-related musculoskeletal disorders.

## Data Availability

Not applicable.
